# Highly Stretchable Electrodes on Wrinkled Polydimethylsiloxane Substrates

**DOI:** 10.1038/srep16527

**Published:** 2015-11-20

**Authors:** Jun Tang, Hao Guo, Miaomiao Zhao, Jiangtao Yang, Dimitris Tsoukalas, Binzhen Zhang, Jun Liu, Chenyang Xue, Wendong Zhang

**Affiliations:** 1 Science and Technology on Electronic Test & Measurement Laboratory, Ministry of Education, Shanxi, 030051, China.; 2 Key Laboratory of Instrumentation Science & Dynamic Measurement (North University of China), Ministry of Education, Shanxi, 030051, China; 3 Department of Applied Physics, National Technical University of Athens, GR-15780 Zografou, Greece

## Abstract

This paper demonstrates a fabrication technology of Ag wrinkled electrodes with application in highly stretchable wireless sensors. Ag wrinkled thin films that were formed by vacuum deposition on top of pre-strained and relaxed polydimethylsiloxane (PDMS) substrates which have been treated using an O_2_ plasma and a surface chemical functionalization process can reach a strain limit up to 200%, while surface adhesion area can reach 95%. The electrical characteristics of components such as resistors, inductors and capacitors made from such Ag conductors have remained stable under stretching exhibiting low temperature and humidity coefficients. This technology was then demonstrated for wireless wearable electronics using compatible processing with established micro/nano fabrication technology.

Due to their new and unconventional applications in optoelectronics and sensors, flexible electronics have been well documented. Paperlike displays and touchscreens are the earliest applications of these technologies which are already under exploration for at least 20 years^1^. More recently, researchers have been continuing their efforts on reducing size and weight, while increasing reliability of flexible electronics to widen their application domains towards biomedical systems, electronic skin, robots etc. Someya *et al.*^2^ have proposed an ultra-light weight design of a microelectronic system on flexible substrate with a thickness of 1 μm and weight of 3 g m^−2^, which could greatly minimize electronic waste and be used in the fields of robotics, biomedical systems and mobile electronics^3^. Park *et al.*^4^ have reported flexible bimodal sensor arrays for simultaneous sensing of pressure and temperature, applied in the field of electronic skin^5^. Flexible supercapacitors for self-powered systems were also discussed using carbon nanotube (CNT) fiber or composite materials as the storage dielectric.

Towards more flexible but also stretchable devices, micro/nano electrodes and wires are one of the most important components that require new materials and new mechanical designs^6^. Carbon black based elastic conductors have been investigated but their strain induced resistance variations are still high. Fukushima *et al.*^7^ proposed rubber like stretchable elastic conductors by using SWCNT/PDMS composite that were integrated with a printed organic transistor active matrix. Using this method, the material exhibits good conductivity of 57 siemens per centimeter and a stretchability of 134% which enable the development of intelligent surfaces and particularly of friendly human/electronics interfaces.

To optimize the mechanical structures and increase flexibility as well as stretchability of silicon electronic components, a reduction of the silicon thickness has been proposed. Rogers *et al.*^8^ reported a human-machine interface via epidermal electronics with optimized mechanical structure and geometrical design using Au/Cr electrodes and Si nano membranes which successfully incorporate electrophysiological, temperature and strain sensor, as well as diodes, capacitors and inductors^9^. Another method proposed along these lines is the use of “wavy” shapes of metallic interconnects and their succesful integration onto elastic substrates.

In this paper a fabrication method of highly stretchable metal electrodes on PDMS substrate is presented and discussed. In a first step the PDMS substrate is pre-strained. Then and in order to make the PDMS surface hydrophilic, we have followed a combined modification approach of the PDMS surface by both O_2_ plasma and Sodium Dodecyl Sulfate (SDS) solution followed by Ag deposition by sputtering. We remark that just before applying SDS and depositing the metal, we have relaxed the strain from the PDMS substrate a process that finally resulted in the formation of wrinkled Ag conductors. The Ag conductors thus formed can reach a strain limit up to 200%. By analyzing the metal electrodes stability in temperature and humidity we obtain that the temperature and humidity coefficients remain low for resistors, inductors or capacitors. These components have been then used as part of wireless wearable sensors to sense the strain during the process of finger bending, as a demonstration example of e-skin. Compared with previous attempts to use Ag on pre-strained PDMS^10^ our room temperature process results demonstrate much better uniformity of periodic structures on PDMS without cracks of metal films at a much higher than used before pre-strain level (50% against 100%), allowing a detailed study of the performance of stretchable electronic components and the demonstration of e-skin application. We attribute that to the deposition of the metal film following PDMS relaxation and not before^10^,^11^ thus eliminating Poisson’s effect during pre-strain relaxation while the use of a combined plasma treatment with SDS functionalization of the PDMS surface further improves the quality of the metal films.

## Results and Discussion

### Surface functionalization and Ag films deposition

#### Fabrication of Ag stretchable electrodes

In this work we deposit silver as a low resistivity electrode material that has been also previously used for stretchable electrodes fabrication either as an ink or thin film^10^ -or more recently in the form of nanowire network^12^ - as an alternative to gold^11^,^13^. Gutruf *et al.*^13^ have reported that serpentine shape electrodes can sustain much higher strain without forming cracks as it was the case of gold straight electrodes when they have been subjected to higher than gold bulk rupture strain. A similar effect also holds in our experiments since our structure compares well with the serpentine shape electrode design.

**Figure 1:**
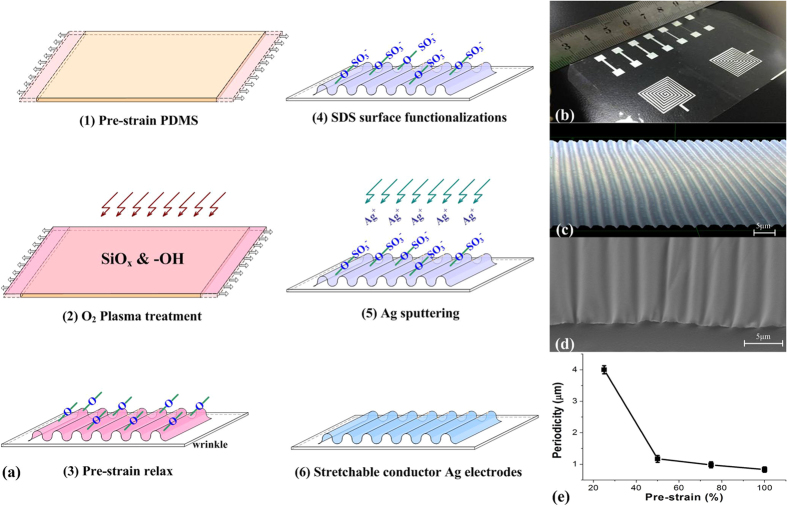
Fabrication process and Morphology characterizations of the stretchable Ag electrodes. (**a**) PDMS treatment and Ag films deposition; (**b**) optical images of the deposited wrinkled electrodes and inductors; (**c**) Laser confocal image of the electrode pattern; (**d**) cross section of the fabricated electrode from an SEM image; (**e**) the periodicity vs the pre-strain.

The fabrication process of the stretchable Ag films is illustrated in Fig. 1a. Oxygen plasma treatment is used to modify the hydrophobic PDMS with hydrophilic functionalities. A SiO_x_ layer and hydrophilic groups (e.g. -OH) have been thus formed on pre-strained PDMS substrates by the O_2_ plasma. When the strain of PDMS exceeds a critical value, the PDMS substrate self-assembles to form folded grating structures after strain relaxation. The PDMS films were then immersed in an SDS solution to introduce -SO_3_^−^ groups at the surface of the wrinkled PDMS grating which can ensure a tight contact between Ag^+^ and PDMS through condensation reactions of hydrophilic functionalities. The role of SDS on PDMS as a functionnalization agent has been discussed in the literature^14^. However the combined use of plasma and SDS treatments has not been investigated before.

As it is shown in Fig. 1c–e, Ag gratings with periodicity from hundreds of nanometers to 10 μm were achieved by tuning the pre-strain and plasma conditions applied^15^. So, different electronic components have been prepared as it is shown in Fig. 1b.

**Figure 2:**
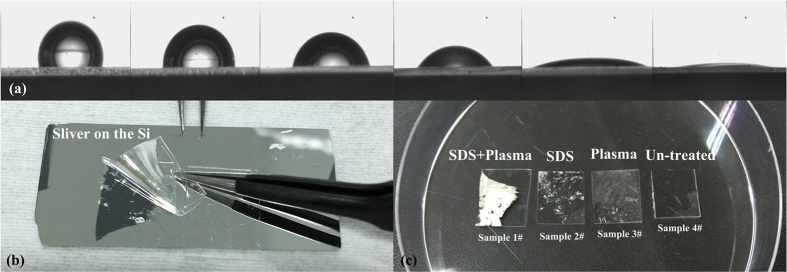
Adhesion test of the treated PDMS surface. (**a**) contact angle measurement of the plasma treated PDMS; (b) Bonding sliver from the Si using the PDMS treated; (**c**) PDMS treated by different methods.

The adhesion force improvement was verified by surface contact angle measurements and surface adhesiveness test. As it is shown in Fig. 2, the contact angle of water against pre-treated PDMS can be observed to be about 10 rad. They both indicate that the adhesion forces were strong enough to bear repetitive scratching.

The purpose of the above double treatment of PDMS surface using O_2_ plasma, followed by a second modification with a surfactant is to render the PDMS surface permanently hydrophilic. As illustrated in ref. 16 reactive species generated by oxygen plasma attacks the siloxane backbone of PDMS to form oxygen rich SiO_x_ silica-like layer and Si-OH compounds on the surface. The main drawback of these modifications is that the oxidized PDMS surface is known to recover its hydrophobicity in days after exposure to air^17^. Plasma treatment improves also the activation properties of inert polymer surface, enhancing the interaction of the interface so that single molecules is more likely to spread onto the surface. The experimental results show that using plasma power at 120 w with processing time of 20 s and a gas flow rate at 150 sccm, the PDMS surface presents no cracks. SDS is an anionic surfactant with amphiphilic properties. Its molecule (CH_3_-(CH_2_)_10_CH_2_O-SO_3_-Na) is composed of lipophilic group of ethyl group CH_3_-CH_2_- that reacts easily with the Si-OH on the surface of PDMS and the hydrophilic group of sulfate ion -O-SO_3_Na is easily hydrolyzed in water. Under conditions of no catalyst and at normal temperature it bonds with Si-O groups at the end of the condensation reaction to form the Si-O-Si-SDS chain that is hydrophilic at the other side. The optimal conditions to obtain an hydrophilic PDMS surface were observed when using a concentration of 0.5% SDS solution for 15 s. Figure 2a shows the contact angle of a water dropplet on the PDMS surface using a Contact Angle Measurement Instrument. We also remark that the method used by plasma to tune the PDMS surface wettability is versatile since the results can be optimized by tuning processing time and power. The SDS solution it is a a way to modify permanently the surface when hydroxyl^18^ groups are present So, in our work we combine these two methods to make the PDMS permanently hydrophilic.

#### Adhesion tests and surface characterization

To characterize adhesion after modification treatment of PDMS, bonding experiments have been performed where we have observed a visual modification effect. These experiments were performed after using various surface treatments in order to acquire more information about the surface adhesion between Ag film and PDMS. As it is shown in Fig. 2b, a silver film is first deposited on a silicon surface. Then a PDMS sample is used to debond the silver film. We have treated the PDMS surface either with plasma or SDS only or with plasma and SDS while an untreated PDMS samle was used as a reference. Comparison btween these four samples show that the combined method of plasma with SDS gives the best results. In Fig. 2c we show that the entire silver film can be removed from the Si surface while adhesion of PDMS is apparently weaker in the other cases.

**Figure 3:**
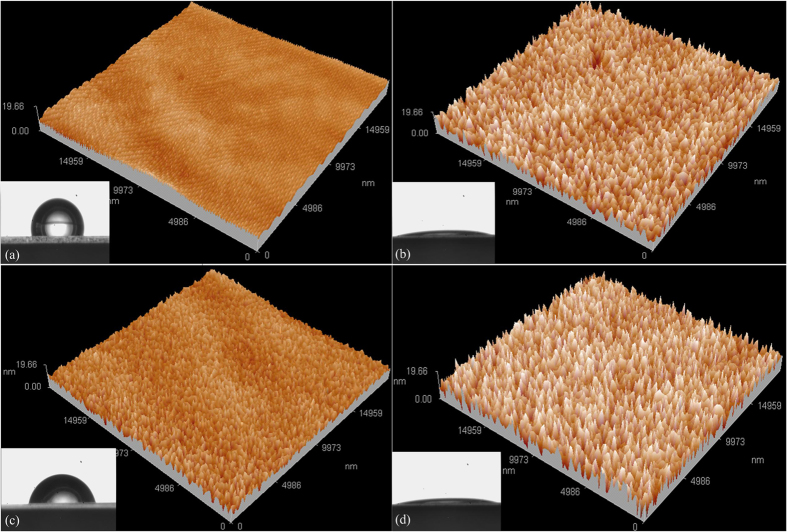
Surface roughness characterizations of various PDMS surfaces. (**a**) as prepared PDMS surface; (**b**) PDMS after 60 s plasma treatment; (**c**) PDMS after 20 s plasma treatment; (**d**) PDMS after plasma (20 s) and SDS joint treatment.

As shown in Fig. 3a, for not treated PDMS there is no modification of surface topography and the surface roughness measured is 0.88 nm. Figure 3b shows that after plasma treatment for long time (60 s) of the PDMS, three-dimensional surface morphology exhibits a lot of bumps and burrs and the roughness becomes 3.44 nm. Figure 3c shows that after plasma treatment for shorter time (20 s) of the PDMS, its surface has some bumps, but the average height of protrusions is lower, while roughness is increased from 0.88 nm to 1.24 nm. Figure 3d shows that by further processing the PDMS of Fig. 3c with a 0.5% SDS surfactant solution for 15 s surface modification progresses and the PDMS surface roughness becomes 3.51 nm.

### Ag electrode patterning and characterization

**Figure 4:**
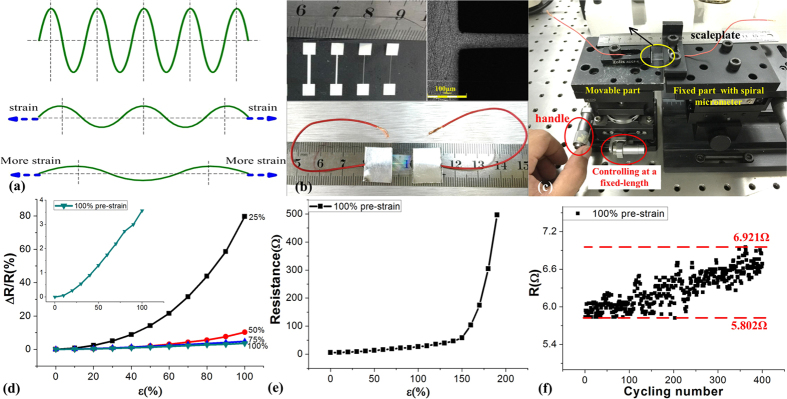
Resistive response characterizations of the wrinkled electrodes. (**a**) stretchable schematic diagram to describe the deformation of wrinkles with applied strain; (**b**) optical image of test structures used and the packaged structures; (**c**) characterization system; (**d**) strain induced resistive performances for samples with different pre-strained (25%–100%); (**e**) strain limitation characterization for the samples with the pre-strain of 100%; (**f**) the reproducibility characterization by stretching cycle test with the sample of 100% pre-strain.

Figure 4a describes qualitatively the deformation of wriknled Ag structures during strain application. Figure 4b shows a series of Ag electrodes and wires which were patterned using a shadow mask with a minimum line width of 50 μm. The resistivity of the film was 8.7 × 10^−9^ Ω·m as calculated by the equation ρ = R*S/L applied to a 10 mm long resistor with linewidth 50 μm and Ag thickness 300 nm. In the above equation R is the initial resistance of 5.802 Ω, S the cross-sectional area of 50 μm*300 nm and L the length of 10 mm. With different pre-strain conditions for preparing the structures, the changes in resistance (ΔR/R, R defined as the initial resistance of the as-prepared sample) of Ag electrodes were studied under stretching with a strain of up to 100%. As shown in Fig. 4c, the samples were fixed to the home-made translation stage which was made by a movable part, a fixed part, a scaleplate, a spiral micrometer (resolution of 10 μm), and a handle & control mechanism. The strain was applied by controlling the micrometer located on the controller, and the repeatability characteristics have been performed by the handle & control mechanism.

As shown in Fig. 4d, the resistance variations depend on the pre-strain applied on the PDMS and they are reduced as the pre-strain is increased. The resistance variations observed were only 4% when the strain has reached a value of 100% (pre-strain was 100%) as shown the inset in the Fig. 4d. No change of the shape of grating structures and the Ag films were observed under stretching and releasing with a strain. Meanwhile, we observe that greater pre-strain values result to smaller grating periods as well as to smaller resistance variations, particularly when the pre-strain is higher than 100%. The strain limitation of our electrodes can reach 200%, as it is determined by the elasticity limit of the polymer matrix (the strain limit of PDMS).

As shown in Fig. 4f, the silver electrodes showed relatively small resistance change in the 400-time cycle test by a cycling test at 100% maximum strain using the sample with the pre-strain of 100%. The normalized resistance slightly increases from an initial resistance of 5.802 Ω to a final resistance of 6.921 Ω after 400-times stretching cycles. It clearly shows that our structures can sustain its initial conductivity under repeated stretching conditions. During a tensile process, the Ag films do not slip and crack, leading to conductance stabilization, owing to a strong adhesion between Ag film and PDMS grating structure.

The nanostructure of the deposited silver film is expected to influence its mechanical and electrical properties. Particularly if the grain size can be tuned by processing mechanical properties be optimized for increased metal performance. A methodology was recently proposed^19^ that makes use of an electrical nanoindentation technique allowing electrical measurements to be performed at the same time with mechanical ones in thin nano-crystalline Pt films. The method after considering various factors that are due to the mechanical force achieves to account for the correct values of hardness and elastic modulus. Such a detailed analysis is beyond the scope of the present study but it could be used in the future to optimize the proposed technique.

### Demonstration of application examples

**Figure 5:**
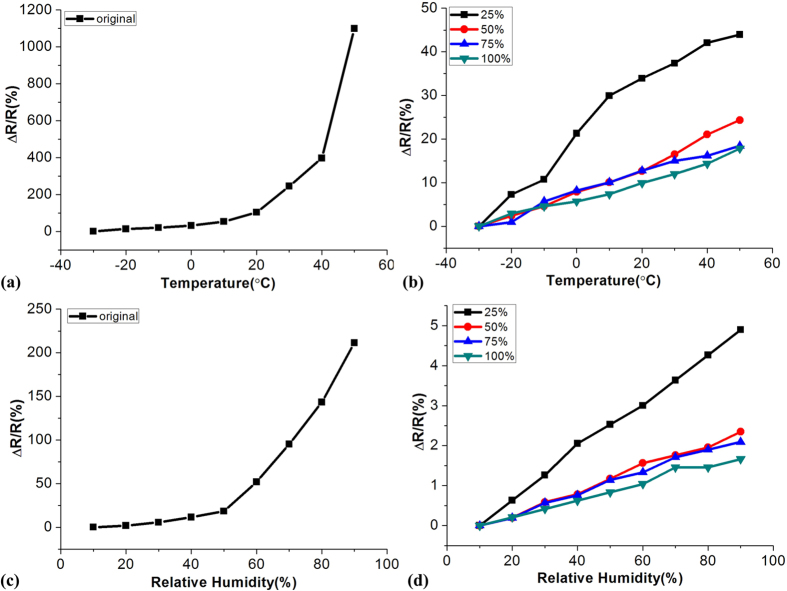
Temperature and humidity charaterizations of the Ag electrodes with and without wrinkled structures. (**a**) Resistance variations of the electrodes as a function of temperature; (**b**) Resistance variations of the electrodes as a function of humidity level.

As shown in Fig. 5, the current exhibits only weak changes under repetitive stretching with strain.

The conductivity of the samples have also been investigated against heating and humidity using the temperature and humidity test chamber with a temperature step of 10 °C (range from −30 °C to 50 °C) and a humidity step of 10 RH% (range from 10 RH% to 90 RH%) respectively. The PDMS is expanding with temperature and humidity increase. However, the change of shape under these environments is much smaller than stretching. From the electrical characterizations, it can also be concluded that the temperature and humidity coefficients of the electrode are 0.002 °C^−1^ and 0.0002 RH%^−1^ respectively (shown in Fig. 5).

**Figure 6:**
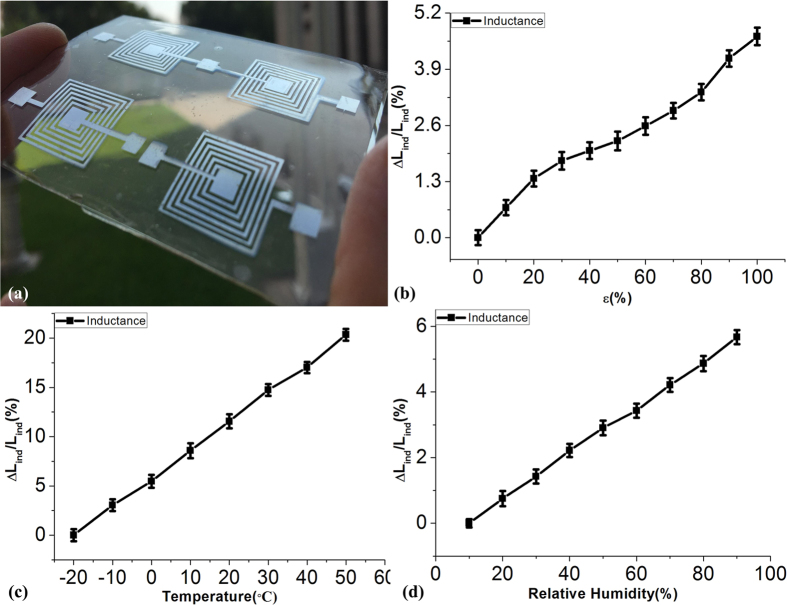
Characterizations of the inductors fabricated by wrinked Ag metals. (**a**) the optical photograph of inductors; (**b**) strain induced inductance varations; (**c**) temperature induced inductance variations; (**d**) humidity induced inductance variations.

Based on the above structure, we have studied and explored also other electronic components. Ten samples with an inductive component (inductors and capacitors) have been prepared using stretchable electrodes with the pre-strain of 100% and their behavior on stretching, temperature and humidity have been tested. As shown in Fig. 6a, the inductors were made using of shadow mask used during sputtering of silver on the pre-strained PDMS substrate with a line width and gap of 0.5 mm. Figure 6a is the optical images of the inductors with a nominal value at 2.233 μH. Figure 6b is the relative inductance change of an inductor structure under 0 to 100% strain effect “(ΔL_ind_/L_ind_, L_ind_ being the initial inductance of the as-prepared sample). As it can be seen the inductance change is less than 5%. Figure 6c,d show that the temperature coefficient of the inductor structure is less than 0.003 °C^−1^ and the relative humidity factor is less than 0.00075 RH%^−1^. We can see that the change due to the stretching and the expansion process is small, the wire structure damage is very limited and the electrical conductivity is still good, so the overall performance of the inductor is satisfactory, definitively demonstrating the buffering effect of the periodic grating structure to streching.

**Figure 7:**
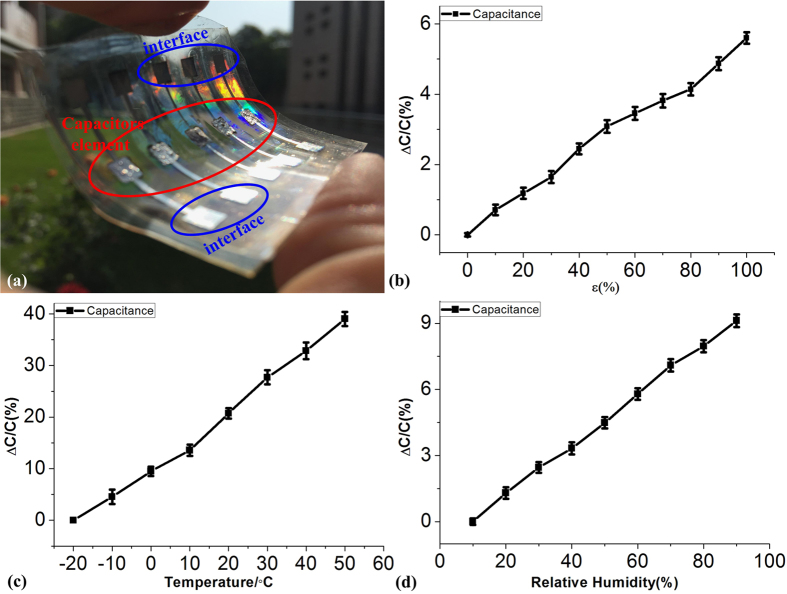
Characterizations of the capacitors fabricated on the winked Ag metals using a CNT/PDMS composite. (**a**) the optical photograph of capacitors; (**b**) strain induced capacitance varations; (**c**) temperature induced capacitance variations; (**d**) humidity induced capacitance variations.

Similarly, as shown in Fig. 7, we have used a stretchable electrode structure in a capacitor with CNTs/PDMS composite as the intermediate dielectric layer. First, the one of the electrodes of capacitor was deposited using a shadow mask on the pre-strained PDMS substrate with a pad area of 3 mm*3mm and a linewidth of the connecting wire 0.5 mm. Second, the CNTs/PDMS composite material was deposited on the pad as the strain sensitive element. Finally, using the same technology as in the first step, the other electrodes were formed on the other side of the CNTs/PDMS composite material to define the capacitors as shown in Fig. 7a, with a nominal value of 1.035 pF. Figure 7b is the relative capacitance change of a capacitor under 0 to 100% strain effect. As it can be seen the capacitance value changes less than 5%. Figure 7c,d represent the temperature coefficient of capacitance structure that is less than 0.006 °C^−1^ in the temperature range −20 °C ~ 50 °C and the relative humidity factor that is less than 0.0012 RH%^−1^ in the relative humidity range 10 RH% ~ 90 RH%. We can see that the change is small and a capacitor with stable capacitance performance is obtained.

Bionic electronic skin^20^,^21^ is a wearable electronic sensor device that can be attached to the body surface to achieve information sensing and transmission. Its simple structure, shape and superior performance can be used for intelligent machines, biomedical, defense and military fields, and has broad application prospects^22^,^23^. CNTs/PDMS composites exhibit high strain-sensitivity, with sensitivity factor as high as 44 that has been already widely applied to flexible electronics, such as bionic electronic skin^24^,^25^, flexible display technology^26^, intelligent robot^27^.

**Figure 8:**
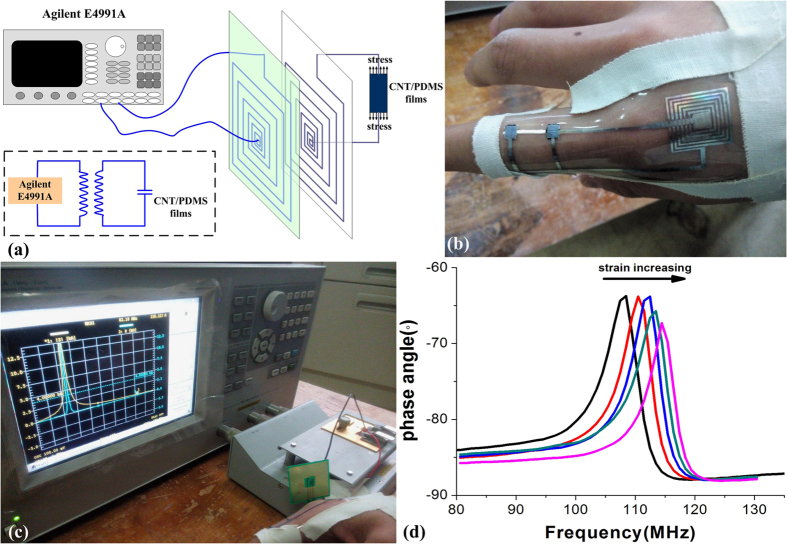
Wearable wireless sensor application test. (**a**) test system configuration; (**b**) sensors on the finger; (**c**) test system; (**d**) test results.

We have then combined our previous studies, together with CNT/PDMS composite used as strain-sensitive unit. Along these lines we have developed a passive wireless oscillation sensor structure and built a sensor test circuit, as it is shown in Fig. 8. With this design, highly strain sensitive capacitors from CNT/PDMS composite combined with highly strain non-sensitive metal electrodes and inductors, which formed an LC circuit, are made as it is shown in Fig. 8a.

The capacitor was fixed onto the finger to measure the bending, so, when fingers bent changes of the capacitance are observed resulting in the oscillation frequency shift of the sensor structure, as it is shown in Fig. 8c. In our work, the finger was bent from the straight state (0°) to the bent state (90°) by a step of about 20°. With this experiment, a frequency shift of the LC oscilation of up to 7.5 MHz has been observed when the bending has changed from 0° to 90° as shown in Fig. 8d. This proved to be a highly senstive wireless method using the strain gauge method with a potential to be further explored in the field of bionic skin applications.

## Conclusions

In summary, highly stretchable Ag films were fabricated after deposition by sputtering on the wrinkles of a pre-strained and relaxed PDMS substrate which was first treated by O_2_ plasma and then surface functionalized using SDS to finally form well adhered Ag gratings used as electrodes with a linewidth of 50 μm.

The resistance variations of Ag stretchable electrodes were only 4% when the strain reached a value of 100%. The stretchability and endurance of our structures are limited by the polymer matrix. It was also demonstrated that the Ag stretchable electrodes are feasible to be used as connecting wires for stretchable circuits.

Along these lines resistors, inductors as well as capacitors have been tested against temperature and humidity variations and results show low corresponding coefficients. These components have been then used in a demonstrative example of a wireless wearable sensor sensing strain during the process of finger bending.

Furthermore, these stretchable electrodes are easy to fabricate and compatible with conventional micro/nano fabrication technology, it can thus be widely used not only for interconnects but also as electrodes for flexible electronics technology, such as electronic skin, flexible display technology or other.

## Methods

PDMS (Sylgard 184) was purchased from Dow Corning. The PDMS (10:1) membranes were prepared by spin coating silicon wafers and cured right after spinning at under 80 °C for 2 hours. PDMS substrates, with a thickness of 500 μm have been prepared by controlling the spinning speed. The concentration of Sodium Dodecyl Sulfate (SDS) is 0.5%. For fabrication of CNT/PDMS nanocomposites, the hydrocarbonyl MWCNTs (diameter: 10–20 nm, length: 1–3 μm) were purchased from Chengdu Organic Chemistry Co., Ltd, Chinese Academy of Science. First, hydrocarbonyl MWCNT were dispersed in chloroform through sonication for 8 hours, and then mixed with PDMS solution by sonication for 4 hours. When almost all of the chloroform has been evaporated, the suspension was moved into a vacuum chamber for one hour to finally form a volume concentration of 2%. All the experiments were performed in a cleanroom area with constant temperature of 20 °C and constant relative humidity at 60%.

As described in Fig. 1, wrinkled SiOx layers were formed on the O_2_ plasma treated pre-strained PDMS substrate (IoN Wave 10, PVA-TePla, Germany), which was fixed to the home-made translation stage (as shown in Fig. 4c). The strain was applied by controlling the micrometer located on the controller. A surface functionalization process using SDS was performed on the SiOx surface to improve the surface adhesion force.

The metallic electrodes were deposited in a high vacuum system equipped with a DC magnetron sputtering source (QPrep400, Mantis, England). The DC magnetron sputtering was performed under the following conditions: DC power of 45 w; Ar flow rate of 30 sccm; chamber pressure of 7.5 × 10^−3^ torr; sputtering time of 5min. The thickness of all the Ag electrodes deposited by sputtering was 300 nm.

The topography of the micro/nano gratings were characterized by Laser Scanning Microscope (LSM) (Model: LEXT OLS4100; Co.: Olympus) and Scanning Electron Microscope (SEM) (Model: S-4800; Co.: Hitachi). The contact angle of water against SDS-treated PDMS was observed by the Contact Angle Measurement Instrument (Model: JGW-360A; Co.: Chenghui). The electrical characterizations of the metallic electrodes and strain gauge elements were performed using Agilent 4156C, 4284A and E4991A.

The temperature and humidity characteristics were performed using a temperature and humidity test chamber (Model: R-PTH80SMS15; Co.: RIUKAI INSTRUMENT TECHNOLOGY). The range of temperature is from −70 °C to 180 °C with an error less than 0.5 °C and the range of the humidity is from 10 RH% to 90 RH% with an error less than 2%.

## Additional Information

**How to cite this article**: Tang, J. *et al.* Highly Stretchable Electrodes on Wrinkled Polydimethylsiloxane Substrates. *Sci. Rep.* **5**, 16527; doi: 10.1038/srep16527 (2015).
